# Ephrin (Eph) receptor A1, A4, A5 and A7 expression in human non-small cell lung carcinoma: associations with clinicopathological parameters, tumor proliferative capacity and patients’ survival

**DOI:** 10.1186/1472-6890-14-8

**Published:** 2014-02-04

**Authors:** Constantinos Giaginis, Nikolaos Tsoukalas, Evangelos Bournakis, Paraskevi Alexandrou, Nikolaos Kavantzas, Efstratios Patsouris, Stamatios Theocharis

**Affiliations:** 1 First Department of Pathology, Medical School, National and Kapodistrian University of Athens, 75 M. Asias str., Goudi, Athens GR11527, Greece; 2 Department of Food Science and Nutrition, School of the Environment, University of the Aegean, Myrina Lemnos, Greece; 3 Department of Medical Oncology, 401 General Military Hospital, Athens, Greece

**Keywords:** Ephrin receptors, Non-small cell lung carcinoma, Clinicopathological parameters, Prognosis, Immunohistochemistry

## Abstract

**Background:**

Ephrin (Eph) receptors are frequently overexpressed in a wide variety of human malignant tumors, being associated with tumor growth, invasion, metastasis and angiogenesis. The present study aimed to evaluate the clinical significance of EphA1, A4, A5 and A7 protein expression in non-small cell lung carcinoma (NSCLC).

**Methods:**

EphA1, A4, A5 and A7 protein expression was assessed immunohistochemically in tissue microarrays of 88 surgically resected NSCLC and was analyzed in relation with clinicopathological characteristics and patients’ survival.

**Results:**

Elevated EphA4 expression was significantly associated with low histopathological stage and presence of inflammation (p = 0.047 and p = 0.026, respectively). Elevated EphA7 expression was significantly associated with older patients’ age, presence of fibrosis and smaller tumor size (p = 0.036, p = 0.029 and p = 0.018, respectively). EphA1, A5 and A7 expression were positively associated with tumor proliferative capacity (p = 0.047, p = 0.002 and p = 0.046, respectively). Elevated EphA4, A5 and A7 expression were identified as predictors of favourable patients’ survival at both univariate (Log-rank test, 0 = 0.019, p = 0.006 and p = 0.012, respectively) and multivariate levels (Cox-regression analysis, p = 0.029, p = 0.068 and p = 0.044, respectively).

**Conclusions:**

The present study supported evidence that Ephs may be involved in lung cancer progression, reinforcing their utility as clinical biomarkers for patients’ management and prognosis, as also as potential targets for future therapeutic interventions.

## Background

Ephrin (Eph) receptors constitute the largest sub-family of receptor tyrosine kinases, being divided into two sub-groups, EphA and EphB, based on their ligand-binding-affinity and structure of the extracellular domain [1,2]. Nine EphA (EphA1-8 and Eph10) and five EphB (EphB1-4 and EphB6) receptors have been identified in humans. Their membrane-anchored ligands, the ephrins, are also divided into two sub-groups, ephrins-A and ephrins-B, which preferentially bind to EphA and EphB receptors, respectively [1-4]. Eph/ephrin interactions occur at sites of cell to cell contact, since both molecules are membrane bound, or between plasma membrane clusters (microdomains) which transform into clearly defined signaling centers upon Eph/ephrin complex formation [5]. In this aspect, Ephs and ephrins have been shown to form a vital cell communication system capable of bi-directional signaling [1,2]. To date, Eph/ephrin signaling have been considered to participate in a wide spectrum of developmental processes, being capable of regulating cellular adhesion, migration or chemo-repulsion and tissue/cell boundary formation [4,6].

Beyond their initial role in developmental processes, Ephs and ephrins have also been involved in a broad range of processes directly related with tumor progression and metastasis, including cell attachment and shape, migration and angiogenesis [7-11]. Notably, Ephs have been considered as master regulators capable of either stimulating the activities of oncogenic signaling networks or repressing them, depending on ephrin stimulation and other contextual factors [9]. Several Ephs and/or ephrins are also expressed in both cancer cells and the tumor microenvironment, where they influence tumor properties by enabling aberrant cell-cell communication within and between tumor compartments. Thus, Eph/ephrin system has been considered as attractive targets for drug design, as targeting these molecules could simultaneously inhibit several aspects of tumor growth and progression [7-11].

Lung cancer is the leading cause of cancer mortality in the world, causing more deaths than colorectal, breast and prostate cancers combined [12]. Non small cell lung cancer (NSCLC) is considered aggressive and highly invasive and accounts for approximately 80% of all lung cancer cases [13]. Despite the development of new surgical procedures and chemotherapeutic protocols, the 5-year survival rate remains less than 15% [12,13]. Smoking is the most important risk factor in the development of most pulmonary carcinomas [12,13]. However, even in a smokeless society, it is predicted that lung cancer due to prior exposure to carcinogens will continue to be a major health problem for the future [14,15].

Accumulative clinical evidence has demonstrated that Ephs are overexpressed in a variety of tumors, being associated with clinicopathological parameters crucial for patients’ management and prognosis [7-11,16-19]. However, despite the gradually increasing research in several human malignancies, there is no comprehensive available data so far concerning the clinical significance of Ephs expression in NSCLC. In view of above considerations, the present study aimed to assess immunohistochemically EphA1, A4, A5 and A7 expression in 88 NSCLC patients, in association with clinicopathological parameters and patients’ survival.

## Methods

### Clinical Material

Eighty-eight NSCLC specimens obtained from equal number of patients who underwent surgical resection due to lung cancer were included in this study. Institutional review board approval was obtained to use archived material for research purposes. None of the patients had received chemotherapy or radiation before surgery. Eastern Cooperative Oncology Group (ECOG) performance status scoring system was used to assess patients’ daily activity and capacity. The resected tumors were classified histologically as lung adenocarcinoma in 56 (63.64%) and squamous cell lung carcinoma in 32 (36.36%) cases. The histopathological grading was assessed according to the criteria described in World Health Organization (WHO) [20]. Disease histopathological stage was assessed according to the TNM-system and the criteria of the International Union against Cancer [21,22]. Histological parameters, including presence of necrosis, fibrosis, inflammation and lymphovascular invasion, were evaluated. Clinicopathological characteristics of the cohort study are described in Additional file 1: Table S1 of the supporting information material. Overall patients’ survival times were defined for the present study. Patients were followed-up for a time interval between 3 and 121 months (mean: 25.39, SD: 22.12 months). At the time of the last follow-up, 82 (93.18%) patients had died due to lung cancer, whereas the remaining 6 (6.82%) patients were alive.

### Tissue microarrays (TMAs)

All the archival tissue samples were routinely fixed in formalin and embedded in paraffin wax. Representative tissue areas were marked on standard hematoxylin and eosin stained sections that were cut from the blocks; these corresponding areas were then punched out of the paraffin block using a 2.0-mm punch, and the cores were inserted into a recipient paraffin block.

### Immunohistochemistry

Immunostainings were performed on TMAs using commercially available rabbit polyclonal EphA1 (G-18, sc-925), EphA4 (S-20, sc-921), EphA5 (C-16, sc-927) and EphA7 (C-19, sc-918) IgG antibodies (Santa Cruz Biochemicals, Santa Cruz, CA, USA). Briefly, 4 μm thick tissue sections were dewaxed in xylene and were brought to water through graded alcohols. Antigen retrieval (citrate buffer at pH 6.1 and microwave heating) was then performed. To remove the endogenous peroxidase activity, sections were treated with freshly prepared 0.3% hydrogen peroxide in methanol in the dark, for 30 minutes (min), at room temperature. Non-specific antibody binding was blocked using a specific blocking reagent for rabbit primary antibodies (Sniper, Biocare Medical, Walnut, Creek, CA, USA) for 5 min. The sections were then incubated for 1 hour (h), at room temperature, with primary antibodies, diluted 1:100 in phosphate buffered saline (PBS). After washing three times with PBS, the sections were incubated at room temperature with biotinylated linking reagent (Biocare Medical) for 10 min, followed by incubation with peroxidase-conjugated streptavidin label (Biocare Medical) for 10 min. The resultant immune peroxidase activity was developed in 0.5% 3,3’-diaminobenzidine hydrochloride (DAB; Sigma, Saint Louis, MO, USA) in PBS containing 0.03% hydrogen peroxide for 5 min. Sections were then counterstained with Harris’s hematoxylin and mounted in Entellan (Merck, Darmstadt, Germany). Appropriate negative controls were performed by omitting the primary antibody and/or substituting it with an irrelevant anti-serum (data not shown). As positive control, pancreatic and thyroid cancer tissue sections with known increased Ephs expression were used (data not shown) [18,19].

### Evaluation of immunohistochemistry

Immunostained slides were evaluated by estimating the percentage and the intensity of positive cells per TMA punch by two independent observers (S.T. and P.A.) blinded to the clinical data, with very good inter-observer agreement (*κ =* 0.982, SE: 0.017). At least 2 cores were analyzed per tumor to assess the EphA1, A4, A5 and A7 immunoreactivity. The immunoreactivity of the tumor cells for EphA1, A4, A5 and A7 was scored according to the percentage of EphA1, A4, A5 and A7 positive tumor cells as 0: negative staining- 0-4% of tumor cells positive; 1: 5-24% of tumor cells positive; 2: 25-49% of tumor cells positive; 3: 50-100% of tumor cells positive, and its intensity as 0: negative staining, 1: mild staining; 2: intermediate staining; 3: intense staining. Finally, the expression of EphA1, A4, A5 and A7 was classified as negative/weak; if the total score was 0 or 2 and moderate/high; if the total score was ≥3. In this way, we ensure that each group has a sufficient and more homogeneous number of cases in order to be comparable with the other groups [18,19,23].

### Statistical analysis

Chi-square test was used to assess the associations of EphA1, A4, A5 and A7 protein expression with clinicopathological variables. Survival curves were constructed using the Kaplan-Meier method and the differences between the curves were compared by the log-rank test. A Cox proportional-hazard regression model was developed to evaluate the association between the potential prognostic marker and overall patients’ survival. A p-value less than 0.05 was considered the limit of statistical significance. SPSS for Windows Software was used for all analyses (SPSS Inc., 2003, Chicago, USA).

## Results

**Figure 1:**
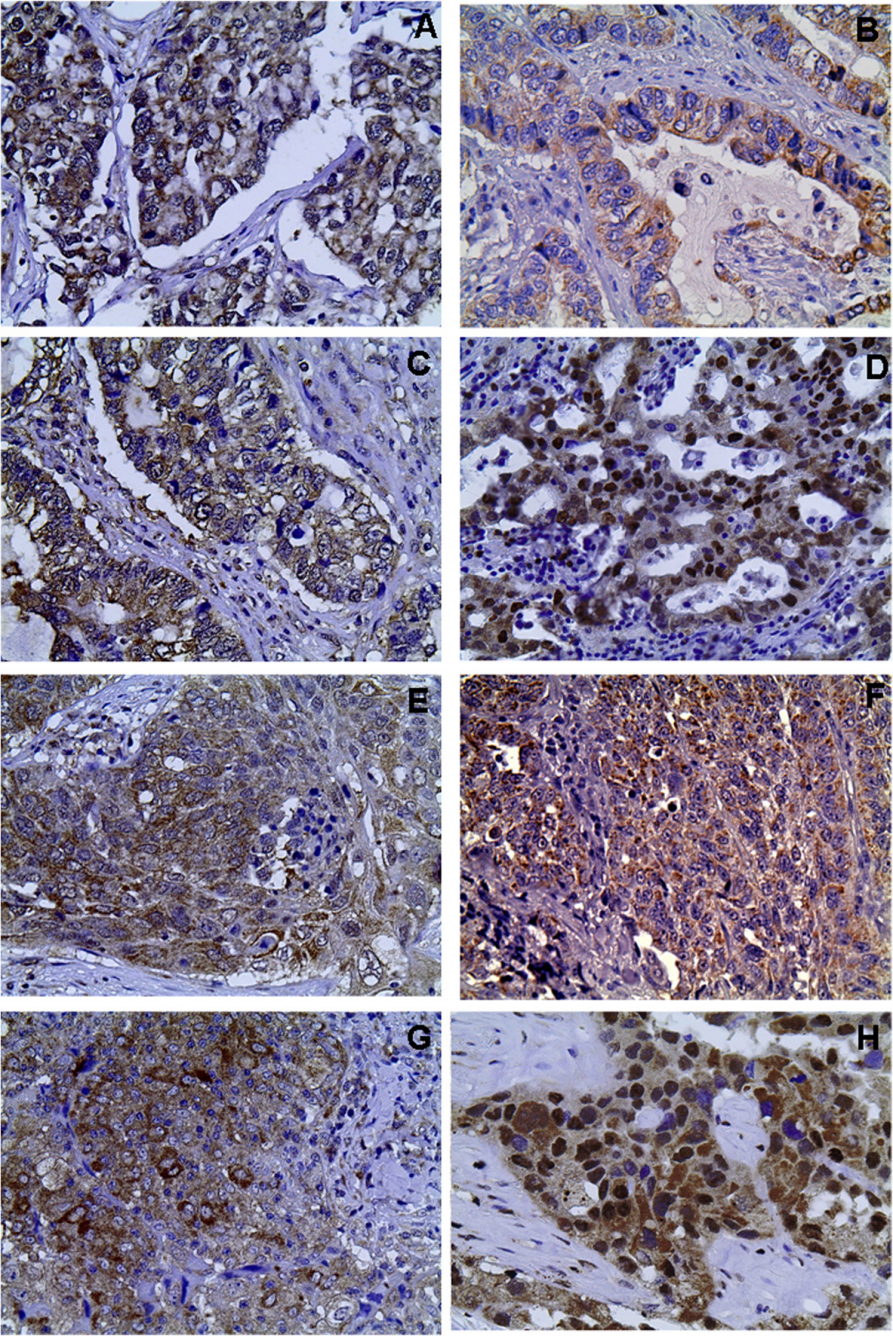
**Representative immunostainings for EphA1, A4, A5 EphA7 protein expression in tumor cells of adenocarcinoma (A, B, C and D, respectively) and of squamous (E, F, G and H, respectively) type NSCLC.** Streptavidin-biotin-peroxidase, DAB chromogen, Harris hematoxylin counterstain (original magnification X400).

Moderate/high EphA1, A4, A5 and A7 expression was noted in 15 (17.0%), 24 (27.3%), 51 (58.0%) and 50 (56.8%) out of 88 NSCLC cases, respectively. Of the 56 adenocarcinoma-type NSCLC cases, moderate/high EphA1, A4, A5 and A7 expression was noted in 9 (16.1%), 13 (23.2%), 35 (62.5%) and 33 (58.9%), respectively. Of the 32 squamous-type NSCLC cases, moderate/high EphA1, A4, A5 and A7 expression was noted in 6 (18.8%), 11 (34.4%), 16 (50.0%) and 17 (53.1%) cases, respectively. EphA1, A4 and A5 presented mainly cytoplasmic and occasionally membraneous pattern of staining, whereas EphA7 showed both cytoplasmic and nuclear pattern of staining (Figure 1).

EphA1 expression did not show significant associations with any clinicopathological parameters examined except for a trend of correlation with lymph node metastases (Table 1). A slightly increased incidence of moderate/high EphA1 expression was observed in NSCLC cases of low histopathological stage, as well as in those presenting absence of distant metastasis (Table 1). Moderate/high EphA4 expression was significantly associated with low stage and presence of inflammation and borderline with lymph node metastases (Table 1). Eph-A5 expression did not show significant associations except for a trend of correlation with patients’ age (Table 2). Moderate/high EphA7 expression was significantly more frequently observed in older patients, as well as in those with presence of fibrosis and smaller tumor size (Table 2). EphA1, A5 and A7 expression was significantly positively associated with tumor proliferative capacity (Tables 1 and 2). Squamous cell lung carcinoma patients showed a no significant increased incidence of moderate/high EphA4 compared to those with adenocarcinoma (Table 1), while moderate/high EphA5 and A7 expression was more frequently observed in lung adenocarcinoma patients compared to those with squamous cell carcinoma (Table 2). A no significant increased incidence of moderate/high EphA5 and A7 expression was also observed in NSCLC patients presenting low stage and well/moderately differentiation (Table 2). Moderate/high EphA1, A4, A5 and A7 expression was more frequently observed in NSCLC patients with ECOG performance status < 2 compared to those with ECOG performance status ≥ 2, at a non significant level (Tables 1 and 2).

**Table 1:** Associations of Eph-A1 and -A4 expression with clinicopathological characteristics in 88 NSCLC patients

**Clinicopathological characteristics**	**Eph-A1 expression**			**Eph-A4 expression**		
	**Negative/weak**	**Moderate/high**	**p-value**	**Negative/weak**	**Moderate/high**	**p-value**
**N = 88**	73 (83.0)	15 (17.0)		64 (72.7)	24 (27.3)	
**Age** (mean ± SD;ys)			0.511			0.486
≤ 64.47 ± 9.18 yrs	36 (40.9)	6 (6.8)		32 (36.4)	10 (11.4)	
> 64.47 ± 9.18 yrs	37 (42.0)	9 (10.2)		32 (36.4)	14 (15.9)	
**Gender**			0.841			0.693
Female	13 (14.8)	3 (3.4)		11 (12.5)	5 (5.7)	
Male	60 (68.2)	12 (13.6)		53 (60.2)	19 (21.6)	
**Histopathological type**			0.748			0.258
Adenocarcimoma	47 (53.4)	9 (10.2)		43 (48.9)	7 (14.8)	
Squamous	26 (29.5)	6 (6.8)		21 (23.9)	11 (12.5)	
**Histopathological grade**			0.569			0.963
I + II	48 (54.5)	11 (12.5)		43 (48.9)	16 (18.2)	
III	25 (28.4)	4 (4.5)		21 (23.9)	8 (9.1)	
**Smoking status**			0.792			0.837
Yes	65 (73.9)	13 (14.8)		57 (64.8)	21 (23.9)	
No	8 (9.1)	2 (2.3)		7 (14.8)	3 (3.4)	
**Alcohol consumption**			0.398			0.503
Systematic	40 (45.5)	10 (11.4)		36 (40.9)	14 (15.9)	
No systematic	33 (37.5)	5 (5.7)		28 (31.8)	10 (11.4)	
**Performance status**			0.453			0.469
0-1	63 (71.6)	14 (15.9)		55 (62.5)	22 (25.0)	
2	10 (11.4)	1 (1.1)		9 (10.2)	2 (2.3)	
**Inflammation**			0.869			0.026
Yes	16 (18.2)	3 (3.4)		10 (11.4)	9 (10.2)	
No	57 (64.8)	12 (13.6)		54 (61.4)	15 (17.0)	
**Lymphovascular invasion**			0.788			0.273
Yes	22 (25.0)	4 (4.5)		21 (23.9)	5 (5.7)	
No	51 (58.0)	11 (12.5)		43 (48.9)	19 (21.6)	
**Necrosis**			0.785			0.253
Yes	41 (46.6)	9 (10.2)		34 (38.6)	16 (18.2)	
No	32 (36.4)	6 (6.8)		30 (34.1)	8 (9.1)	
**Fibrosis**			0.593			0.821
Yes	14 (15.9)	2 (2.3)		12 (13.6)	4 (4.5)	
No	59 (67.0)	13 (14.8)		52 (59.1)	20 (22.7)	
**Tumor size**			0.941			0.700
T1	14 (15.9)	3 (3.4)		13 (14.8)	4 (4.5)	
T2-4	59 (67.0)	12 (13.6)		51 (58.0)	20 (22.7)	
**Lymph node metastases**			0.091			0.062
N0-1	47 (53.4)	13 (14.8)		40 (45.5)	20 (22.7)	
N1-2	26 (29.5)	2 (2.3)		24 (27.3)	4 (4.5)	
**Distant metastases**			0.250			0.120
M0	67 (76.1)	15 (17.0)		58 (65.9)	24 (27.3)	
M1	6 (6.8)	0 (0.0)		6 (6.8)	0 (0.0)	
**Histopathological stage**			0.185			0.047
I + II	40 (45.5)	11 (12.5)		33 (37.5)	18 (20.5)	
III + IV	33 (37.5)	4 (4.5)		31 (35.2)	6 (6.8)	
**Ki-67 protein statement**			0.047			0.104
< mean value	35 (39.8)	3 (3.4)		31 (35.2)	7 (8.0)	
≥ mean value	38 (43.2)	12 (13.6)		33 (37.5)	17 (19.3)	

**Table 2:** Associations of Eph-A5 and -A7 expression with clinicopathological characteristics in 88 NSCLC patients

**Clinicopathological characteristics**	**Eph-A5 expression**			**Eph-A7 expression**		
	**Negative/weak**	**Moderate/high**	**p-value**	**Negative/weak**	**Moderate/high**	**p-value**
**N = 88**	37 (42.0)	51 (58.0)		38 (43.2)	50 (56.8)	
**Age** (mean ± SD;ys)			0.061			0.036
≤ 64.47 ± 9.18 yrs	22 (25.0)	20 (22.7)		23 (26.1)	19 (21.6)	
> 64.47 ± 9.18 yrs	15 (17.0)	31 (35.2)		15 (17.0)	31 (35.2)	
**Gender**			0.333			0.287
Female	5 (5.7)	11 (12.5)		5 (5.7)	11 (12.5)	
Male	32 (36.4)	40 (45.5)		33 (37.5)	39 (44.3)	
**Histopathological type**			0.235			0.597
Adenocarcimoma	21 (23.9)	35 (39.8)		23 (26.1)	33 (37.5)	
Squamous	16 (18.2)	16 (18.2)		15 (17.0)	17 (19.3)	
**Histopathological grade**			0.406			0.499
I + II	23 (26.1)	36 (40.9)		24 (27.3)	35 (39.8)	
III	14 (15.9)	15 (17.0)		14 (15.9)	15 (17)	
**Smoking status**			0.412			0.829
Yes	34 (38.6)	44 (50.0)		34 (38.6)	44 (50.0)	
No	3 (3.4)	7 (8.0)		4 (4.5)	6 (6.8)	
**Alcohol consumption**			0.389			0.489
Systematic	23 (26.1)	27 (30.7)		20 (22.7)	30 (34.1)	
No systematic	14 (15.9)	24 (27.3)		18 (20.5)	20 (22.7)	
**Performance status**			0.369			0.626
0-1	31 (35.2)	46 (52.3)		34 (38.6)	43 (48.9)	
2	6 (6.8)	5 (5.7)		4 (4.5)	7 (8.0)	
**Inflammation**			0.297			0.529
Yes	6 (6.8)	13 (14.8)		7 (8.0)	12 (13.6)	
No	31 (35.2)	38 (43.2)		31 (35.2)	38 (43.2)	
**Lymphovascular invasion**			0.328			0.403
Yes	13 (14.8)	13 (14.8)		13 (14.8)	13 (14.8)	
No	24 (27.3)	38 (43.2)		25 (28.4)	37 (42.0)	
**Necrosis**			0.656			0.540
Yes	20 (22.7)	30 (34.1)		23 (26.1)	27 (30.7)	
No	17 (19.3)	21 (23.9)		15 (17.0)	23 (26.1)	
**Fibrosis**			0.879			0.029
Yes	7 (8.0)	9 (10.2)		3 (3.4)	13 (14.8)	
No	30 (34.1)	42 (47.7)		35 (39.8)	37 (42.0)	
**Tumor size**			0.530			0.018
T1	6 (6.8)	11 (12.5)		3 (3.4)	14 (15.9)	
T2-4	31 (35.2)	40 (45.5)		35 (39.8)	36 (40.9)	
**Lymph node metastases**			0.302			0.179
N0-1	23 (26.1)	37 (42.0)		23 (26.1)	37 (42.0)	
N1-2	14 (15.9)	14 (15.9)		15 (17.0)	13 (14.8)	
**Distant metastases**			0.206			0.229
M0	33 (37.5)	49 (55.7)		34 (38.6)	48 (54.5)	
M1	4 (4.5)	2 (2.3)		4 (4.5)	2 (2.3)	
**Histopathological stage**			0.285			0.378
I + II	19 (21.6)	32 (36.4)		20 (22.7)	31 (35.2)	
III + IV	18 (20.5)	19 (21.6)		18 (20.5)	19 (21.6)	
**Ki-67 protein statement**			0.002			0.046
< mean value	23 (26.1)	15 (17.0)		21 (23.9)	17 (19.3)	
≥ mean value	14 (15.9)	36 (40.9)		17 (19.3)	33 (37.5)	

**Figure 2:**
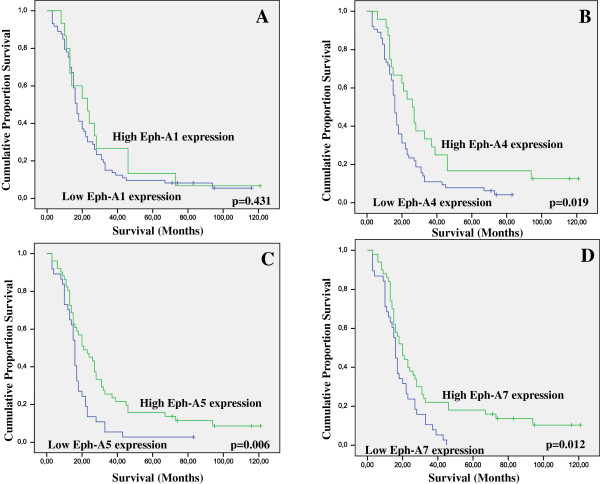
Kaplan-Meier survival analysis stratified according to A. EphA1, B. EphA4, C. EphA5 and D. EphA7 expression in 88 NSCLC patients (–complete cases; + censored cases).

In univariate survival analysis, histopathological type and stage, ECOG performance status and EphA4, A5 and A7 expression were identified as significant prognostic factors of patients’ survival (Table 3, Cox regression analysis, p = 0.013, p < 0.001, p = 0.018, p = 0.025, p = 0.012 and p = 0.016, respectively). Kaplan-Meier survival curves indicated that NSCLC patients presenting moderate/high EphA4, A5 or A7 expression showed significantly longer survival times compared to those with negative/weak expression (Figure 2, log-rank test, p = 0.019, p = 0.006 and p = 0.012, respectively). In multivariate analysis, adjusting for histopathological type and stage and ECOG performance status, EphA4 and A7 expression were identified as independent prognostic factors of patients’ survival (Tables 4 and 5, Cox regression analysis, p = 0.029 and p = 0.044, respectively), whereas EphA5 expression did not remain significant (Table 6, Cox regression analysis, p = 0.068).

**Table 3:** Association of clinicopathological parameters and EphA1, A4, A5 and A7 expression with patients’ survival: Univariate analysis

**Clinicopathological variables**	**Overall patients’ survival**	
	**HR (95% CI)**	**p-value**
**Age** (≤64.47 ± 9.18 / > 64.47 ± 9.18 yrs)	1.148 (0.743-1.773)	0.535
**Gender** (Male/Female)	0.823 (0.472-1.437)	0.494
**Histopathological type** (Adenocarcinoma/Squamous)	1.776 (1.127-2.800)	0.013
**Histopathological grade** (I + II/III)	1.492 (0.944-2.360)	0.087
**Smoking status** (Yes/No)	1.331 (0.680-2.608)	0.404
**Alcohol consumption** (Yes/No)	0.942 (0.608-1.461)	0.791
**Performance status** (0–1/2)	2.178 (1.142-4.155)	0.018
**Inflammation** (Yes/No)	1.354 (0.791-2.317)	0.269
**Lymphovascular invasion** (Yes/No)	0.666 (0.414-1.071)	0.094
**Necrosis** (Yes/No)	0.742 (0.477-1.155)	0.186
**Fibrosis** (Yes/No)	0.637 (0.364-1.115)	0.114
**Tumor size** (T1/T2-4)	2.037 (1.121-3.700)	0.020
**Lymph node metastases** (N0-1/N2-3)	2.323 (1.597-3.380)	<0.001
**Distant metastases** (M0/M1)	4.971 (2.057-12.014)	<0.001
**Histopathological stage** (I + II/III + IV)	2.493 (1.576-3.945)	<0.001
**EphA1 expression** (Negative/Weak *vs* Moderate/Strong)	0.798 (0.448-1.423)	0.444
**EphA4 expression** (Negative/Weak *vs* Moderate/Strong)	0.556 (0.334-0.928)	0.025
**EphA5 expression** (Negative/Weak *vs* Moderate/Strong)	0.542 (0.345-0.853)	0.012
**EphA7 expression** (Negative/Weak *vs* Moderate/Strong)	0.574 (0.365-0.901)	0.016

**Table 4:** Cox regression model including histopathological type and stage, performance status and EphA4 expression

**Variables**	**HR (95% CI)**	**p-value**
**Histopathological type** (Adenocarcinoma/Squamous)	2510 (1.543-4.083)	<0.001
**Histopathological stage** (I + II/III + IV)	2.627 (1.629-4.238)	<0.001
**Performance status** (0–1/2)	2.355 (1.212-4.573)	0.011
**EphA4 expression** (Negative/Weak *vs* Moderate/Strong)	0.544 (0.315-0.939)	0.029

**Table 5:** Cox regression model including histopathological type and stage, performance status and EphA7 expression

**Variables**	**HR (95% CI)**	**p-value**
**Histopathological type** (Adenocarcinoma/Squamous)	2.091 (1.303-3.355)	0.002
**Histopathological stage** (I + II/III + IV)	2.779 (1.729-4.466)	<0.001
**Performance status** (0–1/2)	2.336 (1.201-4.543)	0.012
**EphA7 expression** (Negative/Weak *vs* Moderate/Strong)	0.635 (0.401-1.007)	0.044

**Table 6:** Cox regression model including histopathological type and stage, performance status and EphA5 expression

**Variables**	**HR (95% CI)**	**p-value**
**Histopathological type** (Adenocarcinoma/Squamous)	2.096 (1.306-3.366)	0.002
**Histopathological stage** (I + II/III + IV)	2.207 (1.684-4.352)	<0.001
**Performance status** (0–1/2)	2.231 (1.141-4.362)	0.019
**EphA5 expression** (Negative/Weak *vs* Moderate/Strong)	0.642 (0.398-1.034)	0.068

**Figure 3:**
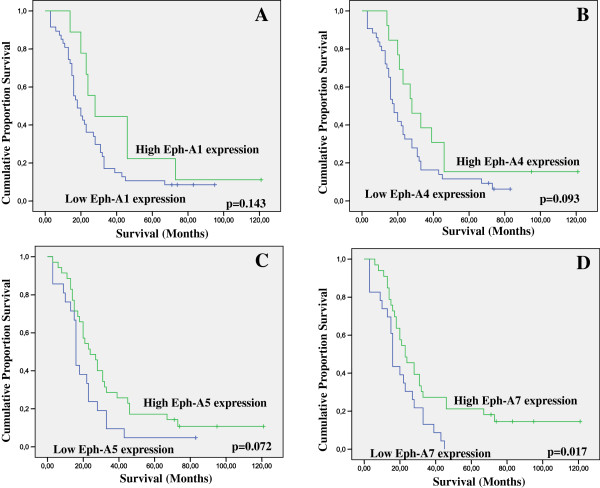
Kaplan-Meier survival analysis stratified according to A. EphA1, B. EphA4, C. EphA5 and D. EphA7 expression in 56 adenocarcinoma-type NSCLC patients (–complete cases; + censored cases).

**Figure 4:**
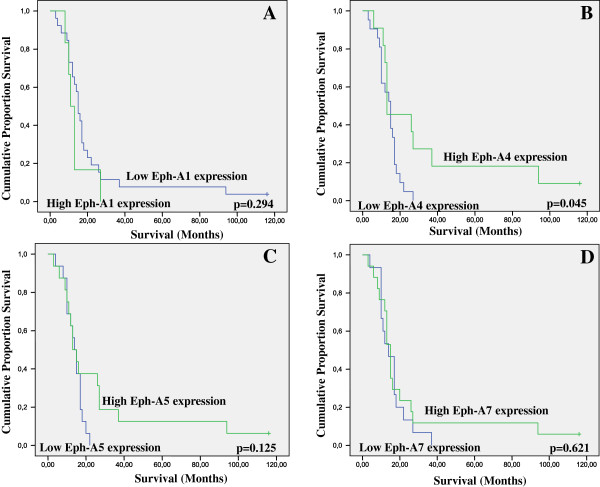
Kaplan-Meier survival analysis stratified according to A. EphA1, B. EphA4, C. EphA5 and D. EphA7 expression in 32 squamous-type NSCLC patients (–complete cases; + censored cases).

Statistical analysis was further performed within each NSCLC histopathological type. EphA1 expression was not associated with either clinicopathological variables (data not shown) or patients’ survival in both subgroups (Figures 3 and 4) except for a borderline association with tumor proliferative capacity in the subgroup of lung adenocarcinoma (p = 0.053).

In the subgroup of lung adenocarcinoma, EphA4 and A5 expression showed trends of correlation with overall patients’ survival (Figure 3, log-rank test, p = 0.093 and p = 0.072, respectively), while EphA7 expression was significantly associated with overall patients’ survival in both univariate (Figure 3, log-rank test, p = 0.017) and multivariate analysis (Cox regression analysis, p = 0.005). EphA4 expression was significantly associated with the presence of inflammation (p = 0.025) and EphA5 expression with tumor proliferative capacity (p = 0.023). EphA7 expression was significantly associated with patients’ age (p = 0.026) and borderline with tumor size (p = 0.085). Moderate/high EphA1, A4, A5 and A7 expression was more frequently observed in lung adenocarcinoma patients with ECOG performance status < 2 compared to those with ECOG performance status ≥ 2, at a non significant level (p > 0.05, data not shown).

In the subgroup of squamous cell lung carcinoma, EphA1, A5 and A7 expression did not affect patients’ survival (Figure 4, log-rank test, p = 0.294, p = 0.125 and p = 0.621, respectively), while no association with ECOG performance status was noted (data not shown). EphA4 expression was significantly associated with overall patients’ survival in univariate (Figure 4, log-rank test, p = 0.045) but not in multivariate analysis after adjustment for histopathological stage (Cox-regression analysis, p = 0.186). EphA5 and A7 expression was significantly positively associated with tumor proliferative capacity (p = 0.006 and p = 0.028, respectively), while EphA4 expression showed a trend of correlation (p = 0.083). EphA7 expression showed trends of correlation with the presence of fibrosis (p = 0.070) and tumor size (p = 0.081).

## Discussion

In the last few years, accumulative evidence has suggested that Ephs and ephrins are frequently overexpressed in a variety of human malignancies, including oesophageal, thyroid, breast, gastric, colon, pancreatic and gynaecological carcinomas, melanomas and neuroblastomas [7-11,16-19]. However, the most comprehensive clinical data so far is restricted to EphA2 receptor and not extending to other members of the Eph family, while the assessment of the clinical significance of Ephs in lung cancer remains still scarce.

In this aspect, the present study supported clinical evidence for possible participation of Ephs in the biological mechanisms underlying the carcinogenic evolution of NSCLC. Although several previous studies have documented the clinical significance of EphA1 in a variety of malignant tumors including, pancreatic, breast, colorectal, urothelial, vulvar and non-melanoma skin carcinoma [16,18,24-27], we did not find any significant associations with clinicopathological parameters and patients’ survival. However, its possible involvement in the biological mechanisms underlying NSCLC should not be excluded, since the increased incidence of moderate/high EphA1 expression observed in low stage NSCLC patients, as well as in those presenting absence of distant metastasis may reach statistical significance in larger cohorts. Notably, we found that elevated EphA4, A5 and A7 expression was significantly associated with favourable prognosis. Elevated EphA4 expression was also significantly associated with low stage and presence of inflammation, while enhanced EphA7 expression with older patients’ age, presence of fibrosis and smaller tumor size. Elevated EphA1, A4, A5 and A7 expression was also associated with increased tumor proliferative capacity. Moreover, the present study showed an elevated EphA1, A4, -A5 and A7 expression in NSCLC patients with ECOG performance status < 2, which reinforces the assumption that such an association may reach statistical significance in larger cohorts. Overall, these findings supported evidence that Eph signaling may be more important in non advanced stages of lung cancer disease.

In line with the present findings, sufficient evidence has recently suggested that Ephs are implicated in lung tumor cell biology [28]. In fact, immunohistochemical analysis on 279 NSCLC cases indicated that elevated EphA2 expression was associated with K-Ras mutations, EGFR activation, smoking history and poor prognosis [29]. Enhanced EphA2 levels were also reported in patients who subsequently developed brain metastases, whereas reduced EphA2 levels identified patients who did not relapse or who developed contralateral lung metastasis [30]. Moreover, elevated EphA2/ephrin-A1 expression was associated with female gender, reduced smoking status, adenocarcinoma type, well differentiated and p-stage IA NSCLC and EGFR gene mutations [31]. This study also showed that elevated EphA2 mRNA expression in p-stage I NSCLC patients was positively related to improved prognosis [31]. Another study analyzing 11 Ephs and 8 ephrins, indicated that EphA4 and ephrin-A1 gene expression was associated with improved lung adenocarcinoma patients’ outcome, possibly by affecting cancer cell migration and invasion [32]. Moreover, decreased EphB6 expression mRNA and protein levels were reported to be associated with increased risk for metastasis development in NSCLC patients [33]. Several mutations in Eph family members, including EphA3, A5, A7, B1 and B6 were identified in lung adenocarcinoma, while advanced stage tumours presented accumulated more EphA7 mutations compared to low stage tumours [34]. More recently, EphB4 expression was correlated with differentiation, lymph node metastasis and TNM stage in 28 NSCLC patients, while the polymorphism in EphB4 at rs314310 appeared to correspond to protein expression and disease susceptibility [35].

Pre-clinical studies have provided compelling evidence that members of the Eph family and their ligands may promote tumor growth, invasion and metastasis and neovascularization [9,17,36]. Tumor suppressive roles have also been reported for Eph receptors, and ligand-dependent *vs* ligand-independent signaling has been considered as one key mechanism underlying tumor suppressive function as opposed to oncogenic effects [9,17,36]. Thus, Eph receptors and their ligands can switch between contrasting activities by using bidirectional signalling, as well as other signalling modalities to influence cancer cell behaviour [9,36]. Taking into consideration the above notions, our findings supported evidence that Eph receptors may be considered as potential regulators capable of repressing the activities of oncogenic signalling in NSCLC progression, depending on ephs stimulation and/or other contextual factors.

Interestingly, accumulative *in vitro* and *in vivo* evidence has suggested that Ephs and their ligands, may represent promising therapeutic targets in cancer [9,17,28,37]. A variety of strategies are currently under evaluation to interfere with their tumor-promoting effects or enhance their tumor-suppressing effects [9,17,28]. Recently, EphA2/ephrin-A1 system has been considered as potential drug target using multiple approaches, such as agonist antibodies, RNA interference, immunotherapy, virus vector-mediated gene transfer, small-molecule inhibitors and nanoparticles [36]. In addition, the functional cross-talk of EphA2 with other oncogenic alterations along in conjunction with encouraging results from pre-clinical combined studies with chemotherapeutic drugs or molecular therapies has reinforced the utility of combination therapies in targeting Ephs overexpression in cancer [36]. Notably, EphA2 siRNA when used in combination with chemotherapeutic drug paclitaxel was more effective in inhibiting growth of HeyA8 or SKOV3 orthotopic ovarian tumors in mice compared to treatment with the control siRNA and paclitaxel [38]. A combination of EphA2 and focal adhesion kinase (FAK) siRNA resulted in significant decrease in ovarian tumors and tumor microvessel density reduction compared to monotherapy [39]. EphA2 overexpression was also identified as a contributing factor towards the development of resistance to Her2-targeted trastutzumab monoclonal antibody therapy [40]. The cross-talk between EphA2 and EGFR signalling supported evidence that simultaneously inhibiting both EphA2 and EGFR overexpression may provide better anti-tumor response [41].

## Conclusions

The present study supported clinical evidence for possible participation of EphA1, A4, A5 and A7 in the biological mechanisms underlying the carcinogenic evolution of NSCLC. Of even more clinical significance are the data supporting the potential role of EphA4, A5 and A7 members in the pathophysiological aspects of the disease that affect patients’ survival. These findings suggested an important potential role of Ephs pathway signalling in non advanced stage NSCLC. Further research conducted on large cohorts that additionally concern more sensitive techniques is strongly recommended. Future studies investigating the soluble/secreted form of Ephs are also recommended in view of the fact that soluble/secreted forms of these proteins may have a potential to distinguish tumour histopathological type in lung cancer [42]. Understanding the complexity of Ephs participation in NSCLC could contribute to the elucidation of the mechanisms underlining lung cancer progression and metastasis that may in turn support the development of novel anti-cancer therapies targeting Eph/ephrin signalling system in this type of human malignancy.

## Competing interests

All authors declare that they have no competing interests.

## Authors’ contributions

GC participated in the design of the study, in the analysis and interpretation of the data and in drafting the manuscript. NT and EB carried out the immunoassays and collected the data. PA and NK participated in the evaluation of the immunohistochemical data and revising manuscript critically for important intellectual content. EP participated in the design of the study and in the acquisition of funding. ST conceived, designed and coordinated the study and the manuscript drafting. All authors read and approved the final manuscript.

## Pre-publication history

The pre-publication history for this paper can be accessed here:

http://www.biomedcentral.com/1472-6890/14/8/prepub

## Supplementary Material

Additional file 1: Table S1 Clinicopathological characteristics of the cohort study. Click here for file
