# Supplementary material for: Ephrin (Eph) receptor A1, A4, A5 and A7 expression in human non-small cell lung carcinoma: associations with clinicopathological parameters, tumor proliferative capacity and patients’ survival
Source: BMC Clin Pathol. 2014 Feb 4;14:8. doi: 10.1186/1472-6890-14-8 (PMC4234387; doi:10.1186/1472-6890-14-8)
Supplement: Additional file 1: Table S1 — Clinicopathological characteristics of the cohort study. [file 1472-6890-14-8-S1.doc]

**Table 1S:** Clinicopathological characteristics of the cohort study

| **Clinicopathological Characteristics** | **Adenocarcinoma** | **Squamous Cell Carcinoma** |
| --- | --- | --- |
| **N=88** | 56 (63.6) | 32 (36.4) |
| **Age** (mean±SD;ys) |  |  |
| ≤ 64.47±9.18 yrs | 29 (33.0) | 13 (14.8) |
| > 64.47±9.18 yrs | 27 (30.7) | 19 (21.6) |
| **Gender** |  |  |
| Female | 13 (14.8) | 3 (3.4) |
| Male | 43 (48.9) | 29 (33.0) |
| **Histopathological grade** |  |  |
| I | 13 (14.8) | 2 (13.2) |
| II | 29 (33.0) | 15 (17.0) |
| III | 14 (15.9) | 15 (17.0) |
| **SMO** |  |  |
| Yes | 46 (52.3) | 32 (36.4) |
| No | 10 (11.4) | 0 (0.0) |
| **ALC** |  |  |
| Yes | 28 (31.8) | 22 (25.0) |
| No | 28 (31.8) | 10 (11.4) |
| **PS** |  |  |
| 0 | 17 (19.3) | 8 (9.1) |
| 1 | 32 (36.4) | 20 (22.7) |
| 2 | 7 (8.0) | 4 (4.5) |
| **INF** |  |  |
| Yes | 13 (14.8) | 6 (6.8) |
| No | 43 (48.9) | 26 (29.5) |
| **LVI** |  |  |
| Yes | 15 (17.0) | 11 (12.5) |
| No | 41 (46.6) | 21 (23.9) |
| **NEC** |  |  |
| Yes | 27 (30.7) | 23 (26.1) |
| No | 29 (33.0) | 9 (10.2) |
| **FIB** |  |  |
| Yes | 10 (11.4) | 6 (6.8) |
| No | 46 (52.3) | 26 (29.5) |
| **Tumor size** |  |  |
| T1 | 11 (12.5) | 6 (6.8) |
| T2 | 30 (34.1) | 19 (21.6) |
| T3 | 8 (9.1) | 5 (5.7) |
| T4 | 7 (8.0) | 2 (2.3) |
| **Lymph node metastases** |  |  |
| N0 | 11 (12.5) | 6 (6.8) |
| N1 | 27 (30.7) | 16 (18.2) |
| N2 | 16 (18.2) | 10 (11.4) |
| N3 | 2 (2.3) | 0 (0.0) |
| **Distant metastasis** |  |  |
| M0 | 52 (59.1) | 30 (34.1) |
| M1 | 4 (4.5) | 2 (2.3) |
| **Histopathological stage** |  |  |
| I | 4 (4.5) | 2 (2.3) |
| II | 26 (29.5) | 19 (21.6) |
| III | 23 (26.1) | 9 (10.2) |
| IV | 3 (3.4) | 2 (2.3) |
